# Quercitrin Nanocoated Implant Surfaces Reduce Osteoclast Activity In Vitro and In Vivo

**DOI:** 10.3390/ijms19113319

**Published:** 2018-10-25

**Authors:** Alba Córdoba, Nahuel Manzanaro-Moreno, Carme Colom, Hans J. Rønold, Staale P. Lyngstadaas, Marta Monjo, Joana M. Ramis

**Affiliations:** 1 Group of Cell Therapy and Tissue Engineering, Research Institute on Health Sciences (IUNICS), University of the Balearic Islands. Ctra. Valldemossa km 7.5, 07122 Palma de Mallorca, Spain; alba.cordoba@uib.es (A.C.); nauel.manzanero@gmail.com (N.M.-M.); mcarmecolom@gmail.com (C.C.); 2 Instituto de Investigación Sanitaria Illes Balears, 07010 Palma de Mallorca, Spain; 3 Oral Research Laboratory and Department for Prosthetic Dentistry, Institute for Clinical Dentistry, University of Oslo, P.O. Box 1109 Oslo, Norway; h.j.ronold@odont.uio.no; 4 Department of Biomaterials, Institute for Clinical Dentistry, University of Oslo, P.O. Box 1109 Oslo, Norway; spl@odont.uio.no

**Keywords:** bone implant interactions, surface chemistry, animal experiments, biomaterials, polyphenols

## Abstract

In this study, the effect on osteoclast activity in vitro and in vivo of titanium implants that were coated with quercitrin was evaluated. Titanium surfaces were covalently coated with the flavonoid quercitrin. The effect of the surfaces on osteoclastogenesis was first tested in vitro on RAW264.7 cells that were supplemented with receptor activator of nuclear factor kappa-B ligand (RANKL) to generate osteoclast-like cells by tartrate-resistant acid phosphatase (TRAP) inmunostaining after five days of culture, and by analysis of the mRNA expression levels of markers related to bone resorption after seven days of culture. A rabbit tibial model was used to evaluate the in vivo biological response to the implant surfaces after eight weeks of healing, analyzing the lactate dehydrogenase (LDH) and the alkaline phosphatase (ALP) activities in the wound fluid that were present at the implant interface and the peri-implant bone mRNA expression levels of several markers related to inflammation, bone resorption and osteoblast-osteoclast interaction. No differences between groups and control surfaces were found in the wound fluid analyses. Moreover, quercitrin implant surfaces significantly decreased the expression of osteoclast related genes in vitro (*Trap*, *CalcR*, *Ctsk*, *H^+^ATPase*, *Mmp9*) and in vivo (*Ctsk*, *H^+^ATPase*, *Mmp9*) as well as the expression of *RankL* in vivo. Moreover, quercitrin surfaces were not cytotoxic for the cells. Thus, quercitrin implant surfaces were biocompatible and decreased osteoclastogenesis in vitro and in vivo. This could be used to improve the performance of dental implants.

## 1. Introduction

Although dental implants present high survival rates, i.e., 94.6% after a follow-up period of at least 10 years [1], the absolute number of dental implant failure becomes significant and causes economic and social impact for patients and dental professionals. The main biological causes of dental implant failure are associated either with a poor osseointegration or with the occurrence of peri-implantitis, especially in patients with a history of periodontal disease [2,3]. Current challenges in dental implant research are focused on successful installation of implants in patients with impaired bone healing, e.g., in elderly populations suffering from diabetes, osteoporosis, radiation therapy or other malconditions. Therefore, there is a need to develop and establish implant surfaces with improved implant-to-bone response [4].

A strategy to improve the biological performance of dental implants, which will almost certainly lead to the third generation of dental implant surfaces, is to modify the implant surface with bioactive molecules with the aim of modulating the host response. In this sense, a lot of research has been done in the last few years, developing and evaluating multiple coatings containing calcium phosphates, extracellular matrix peptides, or growth factors like bone morphogenetic proteins or vascular endothelial growth factors, among others [5,6]. However, none of these surfaces have reached the clinics yet and some concerns have been raised, especially with the use of growth factors, since serious side effects have been reported, mainly caused by the difficult control of the optimum dosage [4]. Anti-resorptive drugs used in the treatment of osteoporosis, like bisphosphonates, have also been explored in implant coatings for patients with poor bone quality [7]. However, the use of bisphophonates faces some reticence due to rare but reported side effects like jaw necrosis or implant failures in patients having dental implants together with systemic administration of bisphosphonates [8].

Flavonoids are a large family of small polyphenolic molecules that are derived from plants, with known antioxidant [9], anti-inflammatory [10] and antibacterial properties [11,12], among other bioactivities. They are present in the daily human diet and their intake has been related to the prevention of cancers, cardiovascular and neurological diseases [13]. Furthermore, flavonoids have shown beneficial effects on the metabolism of bone [14]. Thus, it is of high interest to evaluate flavonoids as potential bioactive molecules for bone therapies since they have a natural origin, are cheap and easily available, do not have side effects and can be easily accepted by the regulatory authorities, therefore decreasing the time to clinics.

Quercitrin (quercetin-3-*O*-rhamnoside) is a yellow coloured flavonoid obtained from tartary buckwheat and from the bark of different species of oak trees [15]. In previous studies from our group, quercitrin showed high biological effects on bone and gingival cells in vitro [16,17] and affected osteoclastic functions decreasing osteoclastogenesis in RAW264.7 cells when administered in solution in vitro [16]. Then, we developed a method to covalently graft quercitrin to titanium surfaces and evaluated the in vitro response on the differentiation of human mesenchymal stem cells to osteoblasts and on human gingival fibroblasts [18,19,20]. Quercitrin-modified Ti surfaces showed promising bone stimulating properties in vitro on human mesenchymal stem cells [18,19], together with higher cell adhesion, anti-inflammatory and extra-cellular matrix boosting effects on human gingival fibroblasts, as well as decreased adhesion of the oral bacterium *Streptococcus mutans* [20]. These results suggested that quercitrin implant surfaces could enhance the performance of dental implants, enhancing the integration of both hard and soft tissue around the implant, increasing the life-time of the implant and decreasing the risk of peri-implantitis. Peri-implantitis is suggested to be the main cause of single crown oral implant failure [2] with a prevalence in the order of 10% for the implants and 20% for the patients up to 5–10 years following implant placement [3].

**Figure 1:**
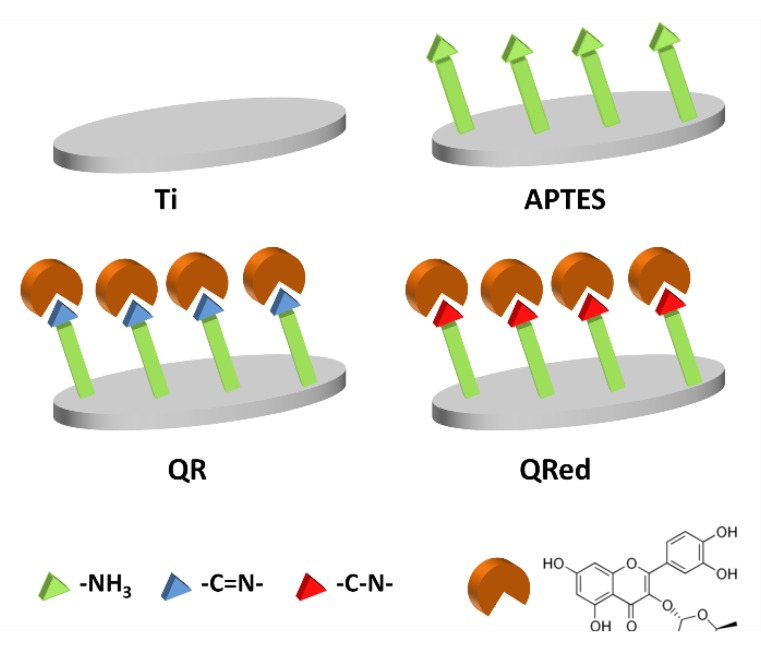
Scheme of the surfaces used in the study. Ti and aminosilanized (APTES) surfaces were used as controls. Quercitrin was covalently grafted to the surfaces in two manners: either by reaction of the carbonyl group of quercitrin with the amino (-NH_3_) terminal group of the aminosilane to give an imine (-C=N-) bond (QR samples) or by adding a reducing agent to the grafting reaction to obtain a single C-N bond between the flavonoid and the silane (QRred samples).

A desirable bioactivity of the third generation of dental implants is to attain a faster formation of the peri-implant bone [6]. This could be accomplished by delaying bone resorption while promoting bone formation, producing more bony tissue just before the remodeling phase. Given the inhibitory effect of quercitrin on osteoclastogenesis [16], we hypothesized here that implant surfaces that are chemically coated with quercitrin would also retain this specific bioactivity. Thus, the aim of the present study was to investigate the effect of quercitrin coated Ti surfaces on osteoclastogenesis in vitro and in vivo in a rabbit tibia model of peri-implant bone healing. For this purpose, four surfaces were evaluated (Figure 1): a Ti control surface and an aminosilanized (APTES) surface were used as controls; and Quercitrin covalently grafted to the surfaces either through the obtention of an imine bond (QR samples) or through the obtention of a C-N bond (QRred samples) between the flavonoid and the silane.

## 2. Results

### 2.1. Effect of the Quercitrin Surfaces on Osteoclastogenesis In Vitro

**Figure 2:**
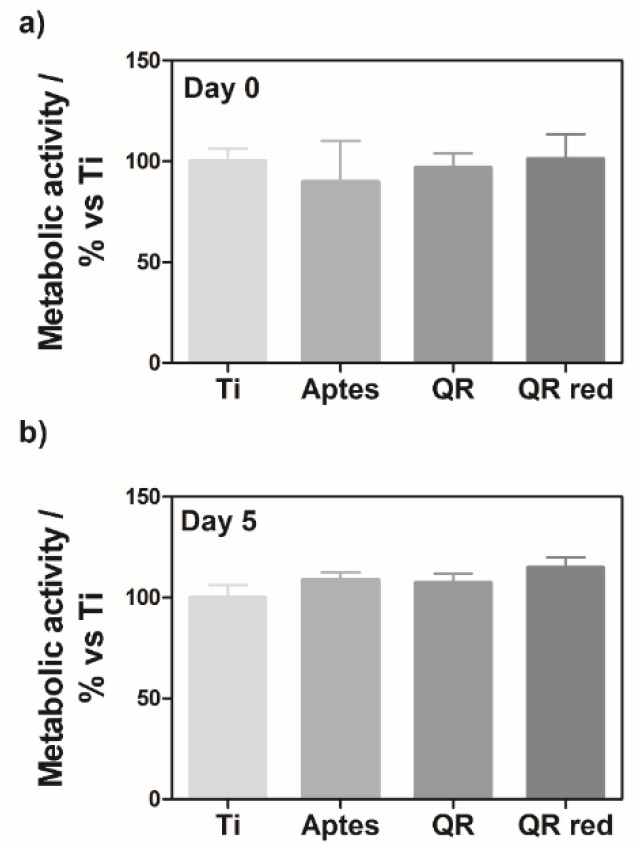
Metabolic activity of RAW264.7 cells cultured on quercitrin coated Ti surfaces was measured at (**a**) day 0 and (**b**) day 5 with a resazurin-based assay (PrestoBlue Cell Viability Reagent). Values represent mean ± SEM, *n* = 6. No significant differences were found between groups as compared by Student-*t*-test.

To assess the effect of the quercitrin implant surfaces on osteoclastogenesis in vitro, RAW264.7 cells were seeded on the different surfaces, and 24 h after seeding the cells, they were treated with RANKL (set as day 0) until day 7 to generate osteoclasts. Cell viability at day 0 and day 5 was determined by measuring the cell metabolic activity by a live-cell assay, showing no significant differences between groups (Figure 2), demonstrating that the surfaces were not cytotoxic for the cells.

**Figure 3:**
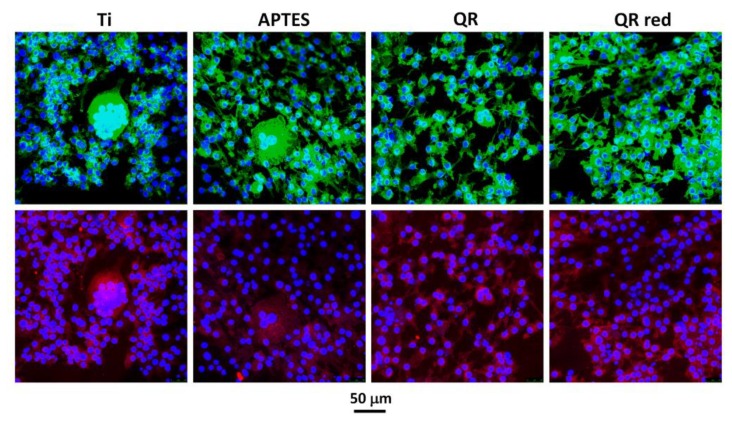
Representative confocal images of multinucleated TRAP (tartrate-resistant acid phosphatase)-positive cells on the different surfaces after 5 days of culture. Cells were stained with Phalloidin-FITC (actin filaments, green), Fluoroshield-DAPI (nucleous, blue) and anti-Trap labeled with Cy3 (Trap protein, red). A higher number of osteoclast-like cells were seen on Ti and APTES surfaces.

After five days of cell culture, the staining of the cells on the surfaces showed the presence of multinucleated TRAP (tartrate-resistant acid phosphatase)-positive osteoclast-like cells on all surfaces (Figure 3). A higher number of osteoclast-like cells (TRAP-positive cells with three or more nuclei) was observed on the control Ti and APTES surfaces.

**Figure 4:**
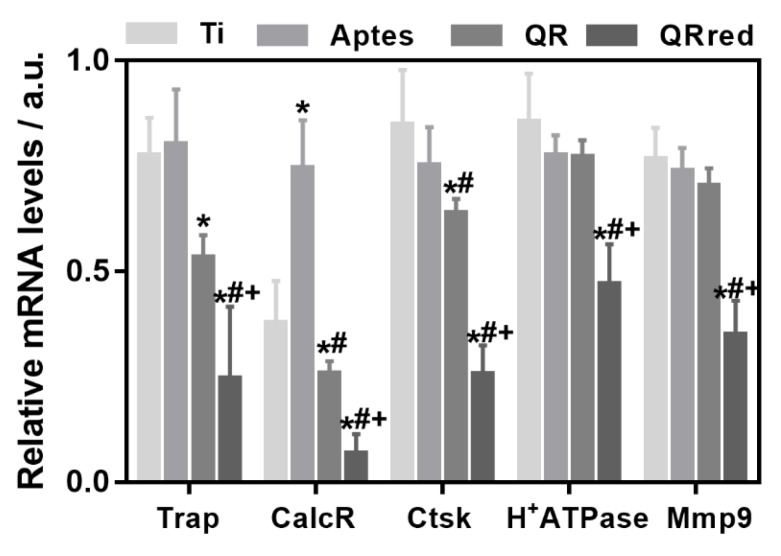
Relative gene expression levels of bone resorption markers after 7 days of culturing RAW264.7 cells in vitro with RANKL (receptor activator of nuclear factor kappa-B ligand). Tartrate-resistant acid phosphatase (*Trap*) and calcitonin receptor (*CalcR*) were analyzed as phenotypic markers, and cathepsin K (*Ctsk)*, vacuolar type proton ATPase (*H^+^ATPase*) and metalloproteinase 9 (*Mmp9*) as functional osteoclastic markers. Values represent mean ± SEM, *n* = 6, a.u. (arbitrary units). *t*-test: * *p* < 0.05 vs. Ti, ^#^ *p* < 0.05 vs. APTES, ^+^ *p* < 0.05 vs. QR.

After seven days of cell culture, the gene expression of phenotypic (*Trap*, *Calc-R*) and functional (*Ctsk*, *H^+^ATPase*, *Mmp9*) osteoclastic markers was evaluated (Figure 4), and we observed that all osteoclastic markers were down-regulated on the cells that were cultured on quercitrin implant surfaces compared to controls. In this way, QR surfaces significantly reduced the expression of *Trap*, *CalcR* and *CtsK*, while QRred surfaces significantly reduced the expression of *Trap*, *CalcR*, *CtsK*, *H^+^ATPase* and *Mmp9*, compared to the control Ti and APTES groups. In all cases, the down-regulating effect of QRred surfaces on the expression of bone resorption related genes was stronger than for QR surfaces. These results indicate that quercitrin coated surfaces decreased osteoclastogenesis in vitro.

### 2.2. Effect of the Quercitrin Surfaces on Osseointegration and Osteoclastogenesis In Vivo

**Figure 5:**
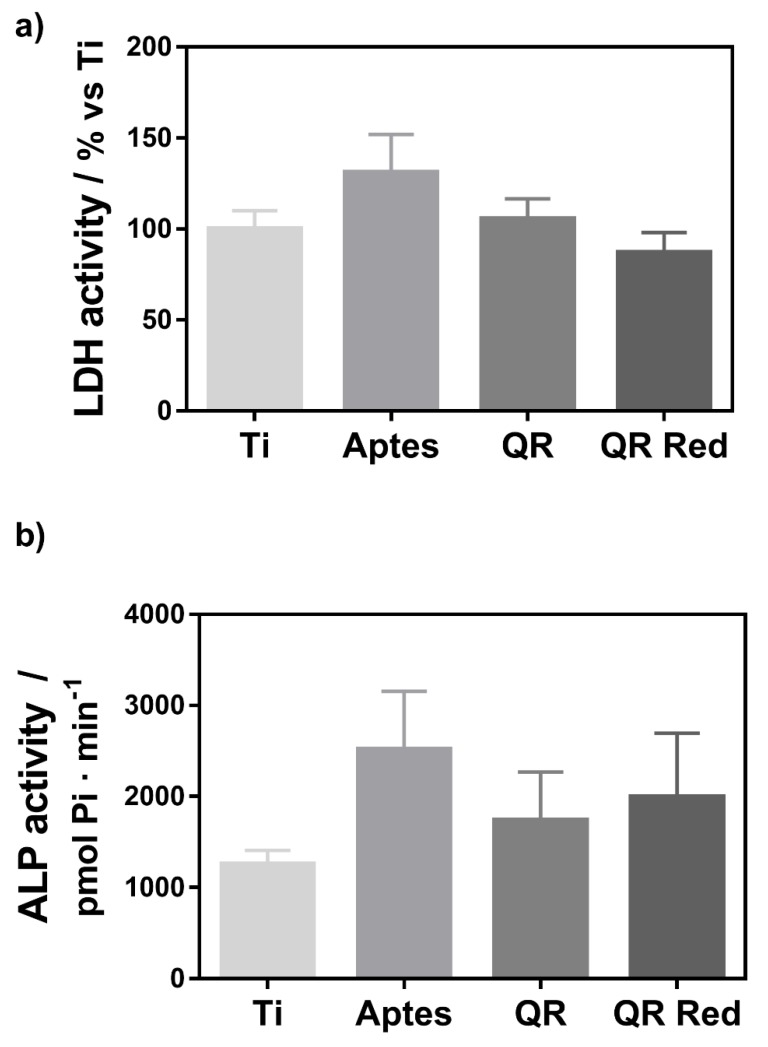
(**a**) LDH (lactate dehydrogenase) activity and (**b**) ALP (Alkaline phosphatase) activity measured in the wound fluid that was collected from the implant site after removing the implant. Values represent mean ± SEM, *n* = 6. No statistical differences were found between the groups.

Figure 5 shows the LDH (lactate dehydrogenase) and ALP (Alkaline phosphatase) activities measured from the wound fluid that was collected from the implant site after the removal of the implants. No significant differences were observed between the groups.

**Figure 6:**
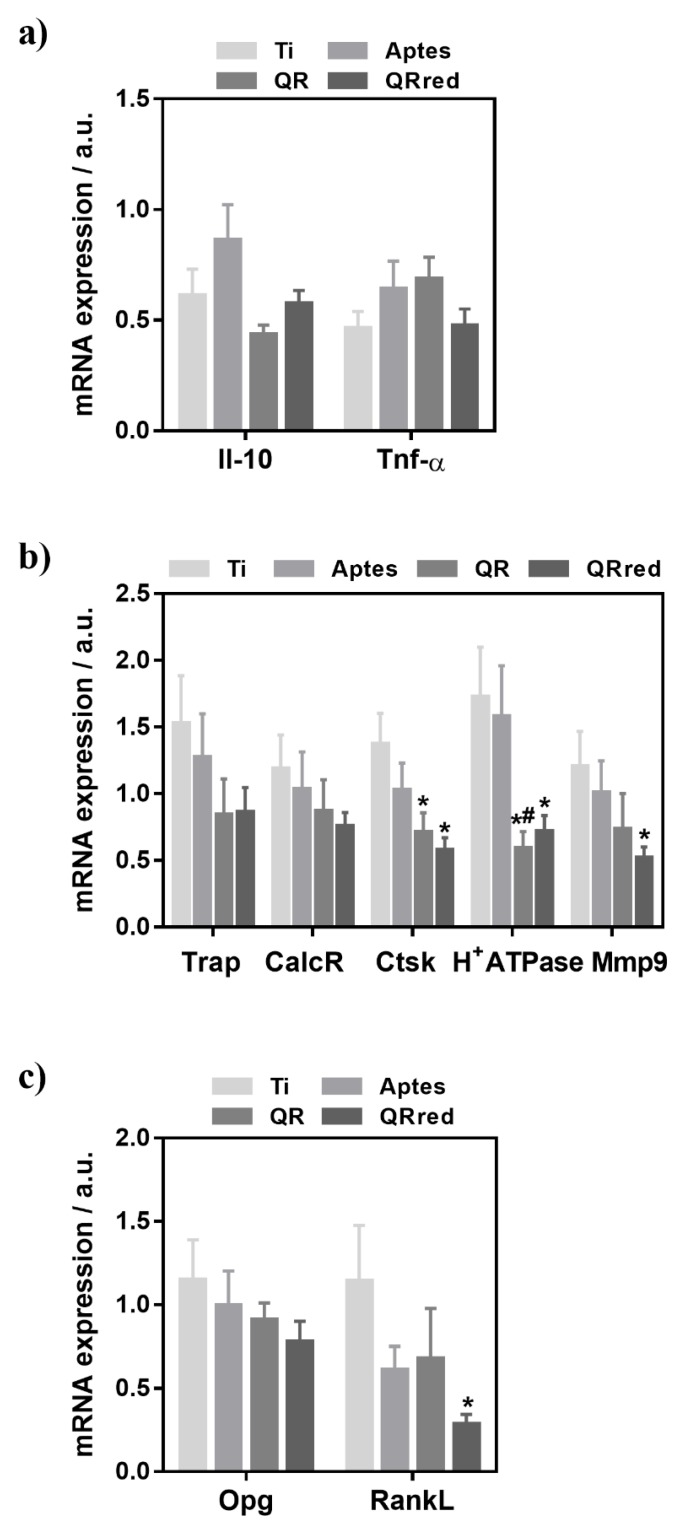
In vivo relative mRNA expression levels of markers related to (**a**) inflammation: the tumor necrosis factor-α (*Tnf-α*) and interleukin 10 (Il-10) (**b**) bone resorption: tartrate-resistant acid phosphatase (*Trap*) and calcitonin receptor (*CalcR*) were analyzed as phenotypic markers, and cathepsin K (*Ctsk*), vacuolar type proton ATPase (*H^+^ATPase*) and metalloproteinase 9 (*Mmp9*) as functional osteoclastic markers and, (**c**) osteoblast-osteoclast interaction: osteoprotegerin (*Opg*) and *RankL*, determined by real time RT-PCR from peri-implant bone tissue on the different quercitrin implant surfaces after 8 weeks of healing. Values represent mean ± SEM, *n* = 6, a.u. (arbitrary units). The *t*-test was performed for all markers except for RankL which was analyzed by the Mann-Whitney test: * *p* < 0.05 vs. Ti, ^#^ *p* < 0.05 vs. APTES.

Figure 6 shows the gene expression analysis from peri-implant bone tissue for bone resorption (*Trap*, *CalcR*, *Ctsk*, *H^+^Atpase*, *Mmp9*), inflammation (*Il-10*, *Tnf-α*) and osteoblast-osteoclast interaction (*Opg* and *RankL*) markers. Regarding inflammation markers, no significant differences were found between the groups. There were no significant differences in the expression levels of phenotypic osteoclastic markers, however the expression of the functional osteoclastic markers *Ctsk*, *H^+^Atpase* and *Mmp9* was significantly lower for quercitrin implant surfaces. Finally, although no differences were found in the *Opg* expression levels between the groups, the expression of *RankL* was significantly lower for QRred surfaces.

## 3. Discussion

Several flavonoid compounds have previously shown to decrease bone resorption [21,22,23,24], also when embedded in polymeric nanoscaffolds [25]. In a previous in vitro study, we observed that quercitrin, administered to cell culture media in micromolar doses, significantly reduced the number of osteoclasts produced from RAW264.7 cells, down-regulated the expression levels of osteoclast related genes *Trap*, *H^+^ATPase*, *Ctsk* and *Mmp9* and decreased *RankL* gene expression in MC3T3 cells [16]. In the present work, titanium surfaces that were covalently coated with quercitrin decreased osteoclast activity both in vitro in RAW264.7 cells that were supplemented with RANKL, and in vivo in a rabbit tibia model, and the effects on mRNA expression levels of the markers related to bone resorption were similar to those that were observed previously when administering the compound in solution in vitro [16]. Therefore, the bioactivity of the flavonoid quercitrin on osteoclastic cells remained, both in vitro and in vivo, when it was covalently linked to a titanium surface. In the in vivo samples, the expression of *RankL* was also significantly lower for QRred surfaces, indicating also an indirect effect of the quercitrin implant surfaces on osteoclast formation in vivo.

The inhibitory effect on the function of osteoclastic cells was higher for QRred surfaces compared to QR surfaces. In previous work, the amount of quercitrin grafted to the surfaces was determined and we found that the amount of quercitrin grafted with the QRred procedure on machined Ti coins was lower (around 0.1 nmol on 6.2 mm coins) than for QR samples (0.2 to 0.8 nmol on the same coins) [19]. Thus, the higher effect of QRred surfaces on osteoclastic cells could be due to the molecular structure of the flavonoid coating, being that the single C-N irreversible bond between the flavonoid and the APTES substrate on QRred samples (Figure 1) is more stable than the imine C=N bond of the QR group.

A main drawback of anti-resorptive molecules, such as biphosphonates, which block osteoclastogenesis, is the presence of osteonecrosis around the implant and the inhibition of osteogenesis [8]. Quercitrin coated surfaces did not decrease pre-osteoclast viability in vitro, as shown by the determinations of cell metabolic activity. Furthermore, in the animal study, no significant differences between groups were found either for LDH or for ALP activities from the wound fluid. The presence of LDH activity in the wound fluid can be used as an index of tissue necrosis, while ALP activity is considered an early mineralization marker and can be negatively correlated with implant osseointegration after an 8-week healing period [26]. Thus, quercitrin coated implants decreased osteoclast function without blocking osteoclastogenesis and without impairing bone formation. The results obtained within the present study agree with other studies evaluating bioactive implant surfaces that enhance bone formation, such as fluoride modified Ti, that did not find differences between the LDH or ALP activities of modified and control groups at this time point using the same animal model [27].

The decrease of osteoclast activity around a dental implant could be beneficial in several aspects. For instance, hydrophilic SLA active surfaces, which are used in clinics with proven success [28,29], have been reported to affect osteoclastogenesis [30,31], decreasing osteoclast differentiation and activity in vitro. Recently, bioinspired dopamine coatings on Ti surfaces had also shown to inhibit the expression of genes related to osteoclast differentiation [32]. A main contribution to dental implant success is related to stability. When an implant is placed, its stability comes from a primary stability given by the mechanical properties of the implant and the existing bone and from a secondary stability due to bone regeneration and remodeling. Hydrophilic SLA active surfaces affect early healing stages, improving the implant stability and allowing earlier loadings [28,29]. As suggested by Bang et al. [30], the decrease in peri-implant osteoclastic activity in hydrophilic SLA active surfaces could contribute to this increased short-term implant stability, as well as to maintain marginal bone levels, increasing also the long term stability of dental implants. Quercitrin implant surfaces could have a similar effect on implant stability. On one side, quercitrin implant surfaces could decrease bone resorption at the bone-implant interface, increasing the implant short-term secondary stability and possibly allowing earlier loadings. On the other side, the inhibition of gene expression of osteoclast functional markers reported here for quercitrin implant surfaces could also probably contribute to maintain marginal bone levels, increasing the implant long-term stability.

In addition, the inhibition of peri-implant osteoclast activity could be beneficial in the case of peri-implantitis, decreasing the bone resorption that is associated with this inflammatory disease, although, in this last case, it should be taken into consideration that an over-inhibition of bone resorption may also help the infection to reach the bone tissue. Since quercitrin implant surfaces did not block osteoclastogenesis, we think that an over-inhibition of bone resorption would be highly improbable for these surfaces.

Finally, quercitrin surfaces could help to increase the success rates of implants that are placed in patients with advanced age who have been reported to suffer higher failure rates [1], having poor bone quality due to an imbalance in the bone resorption process in relation to bone synthesis. However, further studies are needed in hard and soft tissue animal models and clinical trials to confirm these hypotheses.

In conclusion, quercitrin implant surfaces decreased gene expression of osteoclast functional markers both in vitro and in vivo. This could eventually lead to improved performance of quercitrin coated dental implants, possibly allowing early loadings, and contributing to the preservation of the marginal bone level, thus providing long-term stability.

## 4. Materials and Methods

### 4.1. Modification of the Implant Surfaces with Quercitrin

Machined coin-shaped Ti implants, c.p. grade IV, diameter of 6.2 mm and height of 2 mm, were purchased from Implantmedia (Lloseta, Spain) and were cleaned as previously described [33]. For the animal study, the Ti disks were mirror polished (Phoenix 4000, Buehler GmbH, Düsseldorf, Germany) [33] and were then blasted with titanium dioxide (TiO_2_) microparticles to attain an average surface roughness of Sa 1.1 ± 0.1 μm before their modification with quercitrin. Following the TiO_2_ blasting, the Ti coins were cleaned with trichloroethylene in an ultrasonic bath for 30 min, were rinsed with absolute ethanol in an ultrasonic bath for 10 min three times and finally were rinsed with deionised water that was obtained from a Millipore system (Billerica, MA, USA).

All Ti coins were first passivated with 30% HNO_3_ for 30 min, were rinsed thoroughly with water and were left in water overnight (Ti samples in Figure 1). Then, the coins were dried under a N_2_ flow and were immediately immersed in 2% (3-aminopropyl) triethoxysilane (APTES, Sigma Aldrich, St. Louis, MO, USA), an aminosilane used as a coupling compound, in dry toluene for 24 h (APTES samples in Figure 1), were rinsed with dry toluene, acetone and absolute ethanol and were dried under vacuum at 40 °C.

Quercitrin was grafted on the aminosilanized implants in two ways (shown as QR and QRred samples in Figure 1), depending on the grafting reaction conditions. QR samples were obtained as described in reference [18]: the aminosilanized surfaces were chemically functionalized with quercitrin by immersion in a quercitrin hydrate (Sigma Aldrich, St. Louis, MO, USA) 1 mM aqueous solution (250 μL/coin) at pH 5.5 for 1 h at room temperature. In these conditions, the ketone moiety of the flavonoid reacts with the terminal amine group of the silane at mildly acid pH, giving an imine (C=N) bond between the APTES crosslinker and the flavonoid. To obtain QRred samples, passivated and aminosilanized Ti implants were immersed in aqueous solutions of quercitrin hydrate 1 mM (250 L/coin) at pH 7.5, containing sodium cyanoborohydride (NaCNBH_3_, Sigma Aldrich, St. Louis, MO, USA) 100 M for 1 h at room temperature. NaCNBH_3_ selectively reduced the imine bond between the flavonoid and the silane to a single C-N bond [19]. Then, the samples were rinsed twice with water and were dried under a N_2_ flow. All of the samples were prepared under aseptic conditions.

### 4.2. Cell Culture and Generation of Osteoclast-Like Cells In Vitro

The transformed murine monocytic cell line RAW 264.7 was obtained from ATCC (Manassas, VA, USA). The cells were routinely cultured at 37 °C in 5% CO_2_ atmosphere in Dulbecco modified Eagles medium (DMEM) supplemented with 10% fetal bovine serum, 50 IU/mL penicillin and 50 μg/mL streptomycin. The coins were placed in 96-well plates and RAW 264.7 cells were seeded at passage 8 on the different surfaces at a density of 10,000 cells/well. Then, 24 h after seeding, the culture medium was replaced with medium containing 5 ng/mL RANKL (R&D systems, Minneapolis, MN, USA) to generate osteoclast-like cells, and this time-point was set as day 0. Medium containing RANKL was changed every 48 h over the course of 7 days. Cell viability at day 0 and day 5 was determined using a live-cell assay. At day 5, two sample replicates were immunostained for TRAP and at day 7, six sample replicates were used for RNA isolation and gene expression analysis.

### 4.3. Cell Viability

Total metabolic activity was quantified as an indicator of RAW264.7 viability on the different surfaces at day 0 (24 h after seeding) and day 5 using Presto Blue reagent (Life Technologies, Carlsbad, CA, USA) following the manufacturer’s protocols at 1 h of reagent incubation time.

### 4.4. Cell Staining

TRAP-positive multinucleated (three or more nuclei) osteoclasts were visualized by confocal microscopy to confirm the generation of osteoclast-like cells after 5 days of cell culture on the surfaces. Cells on the surfaces were fixed with 2% formaldehyde in phosphate buffer solution (PBS) for 1 h. After removing the formaldehyde solution, bovine serum albumin (BSA) 2% in PBS was added for 30 min. Cells were immunostained overnight at 4 °C with anti-Trap antibody (Thermo Fisher Scientific, Waltham, MA, USA) 1:100 in PBS with 2% BSA, followed by incubation at room temperature with Cy3 (Thermo Fisher Scientific, MA, USA) 1:200 in PBS with 2% BSA for 1.5 h. Between all of the steps, the samples were washed with PBS twice. Cells were then incubated with Phalloidin-FITC 5 μg/mL (Sigma, St. Louis, MO, USA) for 30 min to stain the actin rings. Finally, a drop of Fluoroshield-DAPI (Sigma) was added to stain the cell nuclei and the implants were mounted on microscope slides. Representative images were taken with a confocal microscope (Leica DMI 4000B equipped with Leica TCS SPE laser system). Two implants were observed for each group.

### 4.5. Animal Study

Female New Zealand White rabbits that were 6 months old and weighing 3551 ± 93 g at the surgery and 4004 ± 102 g after the 8-weeks healing period, were used in this in vivo study. The animals were kept in cages during the experimental period. The experiments had been approved and registered by the Norwegian Animal Research Authority (NARA). The procedures were conducted in accordance with the Animal Welfare Act of 20 December 1974, No 73, Chapter VI, Sections 20–22 and the Regulation on Animal Experimentation of 15 January 1996.

Four implants were placed in each animal, with two in each tibia. Six sample replicates were used for each group. Eight weeks after the surgery, the animals were sacrificed and the performance of the implants was analyzed. After removing the implants, two filter papers of the same size as the Ti coins were applied for 1 min in each drilled hole to absorb the wound fluid, then they were transferred to microcentrifuge tubes containing 200 μL of PBS and were placed on ice until analyses were performed the same day. Alkaline phosphatase (ALP) activity was measured from an aliquot of 25 μL of wound fluid in duplicate by measuring the cleavage of p-nitrophenyl phosphate (Sigma, St. Louis, MO, USA) as described in reference [27]. LDH activity of 50 μL of wound fluid was determined spectrophotometrically according to the manufacturer’s kit instructions (Cytotoxicity Detection kit, Roche Diagnostics, Mannheim, Germany). Total protein in the wound fluid for each sample was determined using a BCA protein assay kit from a 15 μL wound fluid aliquot (Pierce, Rockford, IL, USA).

### 4.6. RNA Isolation and Gene Expression Analysis by Real Time RT-PCR

Total RNA of cells that were cultured on the different surfaces for 7 days was isolated using Tripure^®^ (Roche Diagnostics, Mannheim, Germany) according to the manufacturer’s protocol. Total RNA of the peri-implant bone tissue that remained adhered to the implants after their removal after 8 weeks of healing was isolated using Trizol (Invitrogen Life Technologies, Carslbad, CA, USA), according to the manufacturer’s protocol, directly using the implants for RNA extraction.

Briefly, after homogenizing the sample with 1 ml of Trizol or Tripure^®^, 200 µL of chloroform was added and the homogenate was allowed to separate into a clear upper aqueous layer containing RNA after centrifugation at 12,000× *g* for 15 min at 4 °C. Then, RNA was precipitated from the aqueous layer with the addition of 500 µL of isopropanol and centrifugation at 12,000× *g* for 30 min at 4 °C. RNA pellet was then washed with 1 mL of 75% ethanol and was precipitated by centrifugation at 7500 × *g* for 5 min at 4 °C. Finally, RNA pellet was allowed to dry at room temperature and was dissolved with 50 µL of RNAse-free water and was stored at −80 degrees until use for the real-time RT-PCR.

Total RNA was quantified at 260 nm using a Nanodrop spectrophotometer (NanoDrop Technologies, Wilmington, DE, USA). Then, 0.5 μg of total RNA of each in vitro sample and 0.8 μg of each in vivo sample were reverse transcribed to cDNA using a High Capacity RNA-to-cDNA kit (Applied Biosystems, Foster City, CA, USA) following the supplier’s protocol. Real-time PCR was performed using LightCycler FastStart DNA Master PLUS SYBR Green I (Roche Diagnostics, Mannheim, Germany) following the manufacturer’s instructions using specific primers (Table 1 and Table 2).

For the in vitro study, *Trap* and calcitonin receptor (*CalcR*) were analyzed as phenotypic markers [34,35], and cathepsin K (*Ctsk)*, vacuolar type proton ATPase *(H^+^ATPase)* and metalloproteinase 9 (*Mmp9*) were analyzed as functional osteoclastic markers [36,37,38]. For the animal study, the tumor necrosis factor-α (*Tnf-α)* and interleukin 10 *(Il-10)* were assessed as inflammation markers; osteoprotegerin (*Opg)* and *RankL* were assessed as indicators of osteoblast-osteoclast interaction; and, finally, the same genes that were evaluated in the in vitro study were assessed as bone resorption markers (*H^+^ATPase*, *Trap*, *CalcR*, *Ctsk* and *Mmp9*). The samples were normalized by the geometric mean of the expression levels of housekeeping genes *18s rRNA* and *Gapdh* and fold changes were related to the control groups using Equation (1) [39]:

(1) $$\mathrm{Normalized}\;\mathrm{expression}=\frac{E_{\mathrm{target}}{}^{\Delta {\mathrm{Cp}\;}_{\mathrm{target}}\;(\mathrm{mean}\;\text{control-sample})}}{E_{\mathrm{reference}}{}^{\Delta {\mathrm{Cp}\;}_{\mathrm{reference}}\;(\mathrm{mean}\;\text{control-sample})}}$$

where Cp is the crossing point of the reaction amplification curve as determined by the software (Lightcycler 480 software, Roche Diagnostics, Mannheim, Germany) and E is the efficiency of the amplification process.

### 4.7. Statistics

Differences between groups were assessed by Mann-Whitney-test or by Student *t*-test depending on their normal distribution. The Kolmogorov-Smirnov test was used to assume parametric or non-parametric distributions for the normality tests. Software SPSS^®^ v.17.0 (SPSS, Chicago, IL, USA) was used to perform the tests. Results were considered statistically significant at *p*-values ≤ 0.05. Data is shown as values ± standard error of the mean (SEM).

## 5. Patents

A.C., M.M. and J.M.R. are the inventors of a pending patent application based on some aspects of this work (PCT/EP2013/058116).

## Figures and Tables

**Table 1:** Sequence of the specific primers used in the real time RT-PCR analysis of the reference and target genes for the in vitro study.

Gene	Primer Sequence	Product Size (bp)	GeneBank Accession Nr.
*Gapdh*	S 5′-ACC CAG AAG ACT GTG GAT GG-3′ A 5′-CAC ATT GGG GGT AGG AAC AC-3′	171	XM_132897
*18s rRNA*	S 5′-GTA ACC CGT TGA ACC CCA TT-3′ A 5′-CCA TCC AAT CGG TAG TAG CG-3′	151	X00686
*Trap*	S 5′-GCG ACC ATT GTT AGC CAC ATA CG-3′ A 5′-CGT TGA TGT CGC ACA GAG GGA T-3′	144	NM_007388.2
*CalcR*	S 5′-TGG TGC GGC GGG ATC CTA TAA GT-3′ A 5′-AGC GTA GGC GTT GCT CGT CG-3′	150	NM_001042725
*Ctsk*	S 5′-AGC AGA ACG GAG GCA TTG ACT C-3′ A 5′-TTT AGC TGC CTT TGC CGT GGC-3′	92	NM_007802.3
*H^+^ATPase*	S 5′-ACG GTG ATG TCA CAG CAG ACG T-3′ A 5′-CCT CTG GAT AGA GCC TGC CGC A-3′	153	NM_175406.3
*Mmp9*	S 5′-GCT GAC TAC GAT AAG GAC GGC A-3′ A 5′-GCG GCC CTC AAA GAT GAA CGG-3′	114	NM_013599.2

All the primers were of mouse source. S: sense. A: antisense.

**Table 2:** Sequence of the specific primers used in the real time RT-PCR analysis of the reference and target genes for the in vivo study.

Gene	Primer Sequence	Primer Source	Product Size (bp)	GeneBank Accession Nr.
*GAPDH*	S 5′- TGC ACC ACC AAC TGC TTA GC-3′ A 5′- GGC ATG GAC TGT GGT CAT GAG-3′	Human	87	M33197
*18s rRNA*	S 5’-GTA ACC CGT TGA ACC CCA TT-3’ A 5’-CCA TCC AAT CGG TAG TAG CG-3’	Mouse	151	X00686
*IL-10*	S 5′- CCT TTG GCA GGG TGA AGA CT-3′ A 5′- ATG GCT GGA CTC TGG TTC TC-3	Rabbit	175	NM_001082045
*Tnf-α*	S 5′- TCC GTG AAA ACA GAG CAG AA-3′ A 5′- GAG CAG AGG TTC GGT GAT GT-3′	Rabbit	160	NM_001082263
*Opg*	S 5′-TCA TCC AAG ATA TTG ACC TCT GTG A-′3 A 5′-GGGGAGCTGCTCACTTGATT-3′	Rabbit	170	XM_002710603.2
*RANKL*	S 5’- CAG AGC GCA GAT GGA TCC TAA-3′ A 5′- TCC TTT TGC ACA GCT CCT TGA-3′	Human	180	NM_003701.3
*Trap*	S 5′- CCT GGG CGA CAA CTT TTA CT-3′ A 5′- TTG GAG ACC TTG GAA TAG GC-3′	Rabbit	180	NM_001081988
*Calc-R*	S 5′- CAA ATG ACA CCC ATC CAA CA-3′ A 5′- ACA TCC ATC CAT CCC AGG TC-3′	Rabbit	162	NM_001082375.2
*Ctsk*	S 5′-GAC ACC CAG TGG GAG CTA TG-3′ A 5′-TCT TCA CTG GTC ATG TCC CC-3′	Rabbit	188	NM_001082641.1
*H^+^ATPase*	S 5′ -CCG AAA CCT CCT GAA GAA AA-3′ A 5′- ATA GCC GTG GTG CTG AAG TC-3′	Rabbit	165	NM_000600.3
*Mmp9*	S 5′-CAA GGA TGG GAG GTA CTG GC-3′ A 5′-TGT GTA CAC CCA CAC TTG GC-3′	Rabbit	172	NM_001082203.1

S: sense. A: antisense.

## References

[1] Moraschini V., Poubel L.A., Ferreira D.C., Barboza V.F., Barboza Edos S. (2015). Evaluation of survival and success rates of dental implants reported in longitudinal studies with a follow-up period of at least 10 years: A systematic review. Int. J. Oral Maxillofac. Surg..

[2] Jung R.E., Pjetursson B.E., Glauser R., Zembic A., Zwahlen M., Lang N.P. (2008). A systematic review of the 5-year survival and complication rates of implant-supported single crowns. Clin. Oral Implants Res..

[3] Mombelli A., Müller N., Cionca N. (2012). The epidemiology of peri-implantitis. Clin. Oral Implants Res..

[4] Junker R., Dimakis A., Thoneick M., Jansen J.A. (2009). Effects of implant surface coatings and composition on bone integration: A systematic review. Clin. Oral Implants Res..

[5] Bauer S., Schmuki P., von der Mark K., Park J. (2013). Engineering biocompatible implant surfaces. Part I: Materials and surfaces. Prog. Mater. Sci..

[6] Palmquist A., Omar O.M., Esposito M., Lausmaa J., Thomsen P. (2010). Titanium oral implants: Surface characteristics, interface biology and clinical outcome. J. R. Soc. Interface.

[7] Abtahi J., Tengvall P., Aspenberg P. (2012). A bisphosphonate-coating improves the fixation of metal implants in human bone. A randomized trial of dental implants. Bone.

[8] Madrid C., Sanz M. (2009). What impact do systemically administrated bisphosphonates have on oral implant therapy? A systematic review. Clin. Oral Implants Res..

[9] Amić D., Davidović-Amić D., Beslo D., Rastija V., Lucić B., Trinajstić N. (2007). SAR and QSAR of the antioxidant activity of flavonoids. Curr. Med. Chem..

[10] Koeberle A., Werz O. (2014). Multi-target approach for natural products in inflammation. Drug Discov. Today.

[11] Cushnie T.P.T., Lamb A.J. (2011). Recent advances in understanding the antibacterial properties of flavonoids. Int. J. Antimicrob. Agents.

[12] Daglia M. (2012). Polyphenols as antimicrobial agents. Curr. Opin. Biotechnol..

[13] Middleton E., Kandaswami C., Theoharides T.C. (2000). The effects of plant flavonoids on mammalian cells: Implications for inflammation, heart disease, and cancer. Pharmacol. Rev..

[14] Welch A.A., Hardcastle A.C. (2014). The effects of flavonoids on bone. Curr. Osteoporos. Rep..

[15] Fabjan N., Rode J., Kosir I.J., Wang Z., Zhang Z., Kreft I. (2003). Tartary buckwheat (*Fagopyrum tataricum* Gaertn.) as a source of dietary rutin and quercitrin. J. Agric. Food Chem..

[16] Satué M., Arriero M.D.M., Monjo M., Ramis J.M. (2013). Quercitrin and Taxifolin stimulate osteoblast differentiation in MC3T3-E1 cells and inhibit osteoclastogenesis in RAW 264.7 cells. Biochem. Pharmacol..

[17] Gómez-Florit M., Monjo M., Ramis J.M. (2014). Identification of Quercitrin as a Potential Therapeutic Agent for Periodontal Applications. J. Periodontol..

[18] Córdoba A., Satué M., Gómez-Florit M., Hierro-Oliva M., Petzold C., Lyngstadaas S.P., González-Martín M.L., Monjo M., Ramis J.M. (2015). Flavonoid-Modified Surfaces: Multifunctional Bioactive Biomaterials with Osteopromotive, Anti-Inflammatory, and Anti-Fibrotic Potential. Adv. Healthc. Mater..

[19] Córdoba A., Monjo M., Hierro-Oliva M., González-Martín M.L., Ramis J.M. (2015). Bioinspired Quercitrin Nanocoatings: A Fluorescence-Based Method for Their Surface Quantification, and Their Effect on Stem Cell Adhesion and Differentiation to the Osteoblastic Lineage. ACS Appl. Mater. Interfaces.

[20] Gomez-Florit M., Pacha-Olivenza M.A., Fernández-Calderón M.C., Córdoba A., González-Martín M.L., Monjo M., Ramis J.M. (2016). Quercitrin-nanocoated titanium surfaces favour gingival cells against oral bacteria. Sci. Rep..

[21] Kim T.-H., Jung J.W., Ha B.G., Hong J.M., Park E.K., Kim H.-J., Kim S.-Y. (2011). The effects of luteolin on osteoclast differentiation, function in vitro and ovariectomy-induced bone loss. J. Nutr. Biochem..

[22] Xiao F., Zhai Z., Jiang C., Liu X., Li H., Qu X., Ouyang Z., Fan Q., Tang T., Qin A. (2015). Geraniin suppresses RANKL-induced osteoclastogenesis in vitro and ameliorates wear particle-induced osteolysis in mouse model. Exp. Cell Res..

[23] Ming L.-G., Chen K.-M., Xian C.J. (2013). Functions and action mechanisms of flavonoids genistein and icariin in regulating bone remodeling. J. Cell. Physiol..

[24] Hsieh T.-P., Sheu S.-Y., Sun J.-S., Chen M.-H. (2011). Icariin inhibits osteoclast differentiation and bone resorption by suppression of MAPKs/NF-κB regulated HIF-1α and PGE2 synthesis. Phytomedicine.

[25] Ji Y., Wang L., Watts D.C., Qiu H., You T., Deng F., Wu X. (2014). Controlled-release naringin nanoscaffold for osteoporotic bone healing. Dent. Mater..

[26] Monjo M., Ramis J.M., Rønold H.J., Taxt-Lamolle S.F., Ellingsen J.E., Lyngstadaas S.P. (2013). Correlation between molecular signals and bone bonding to titanium implants. Clin. Oral Implants Res..

[27] Monjo M., Lamolle S.F., Lyngstadaas S.P., Rønold H.J., Ellingsen J.E. (2008). In vivo expression of osteogenic markers and bone mineral density at the surface of fluoride-modified titanium implants. Biomaterials.

[28] Oates T.W., Valderrama P., Bischof M., Nedir R., Jones A., Simpson J., Toutenburg H., Cochran D.L. (2007). Enhanced implant stability with a chemically modified SLA surface: A randomized pilot study. Int. J. Oral Maxillofac. Implants.

[29] Ganeles J., Zöllner A., Jackowski J., ten Bruggenkate C., Beagle J., Guerra F. (2008). Immediate and early loading of Straumann implants with a chemically modified surface (SLActive) in the posterior mandible and maxilla: 1-year results from a prospective multicenter study. Clin. Oral Implants Res..

[30] Bang S.M., Moon H.J., Kwon Y.D., Yoo J.Y., Pae A., Kwon I.K. (2014). Osteoblastic and osteoclastic differentiation on SLA and hydrophilic modified SLA titanium surfaces. Clin. Oral Implants Res..

[31] Lotz E.M., Berger M.B., Schwartz Z., Boyan B.D. (2018). Regulation of osteoclasts by osteoblast lineage cells depends on titanium implant surface properties. Acta Biomater..

[32] Ma T., Ge X.-Y., Hao K.-Y., Zhang B.-R., Jiang X., Lin Y., Zhang Y. (2017). Simple 3,4-Dihydroxy-l-Phenylalanine Surface Modification Enhances Titanium Implant Osseointegration in Ovariectomized Rats. Sci. Rep..

[33] Lamolle S.F., Monjo M., Lyngstadaas S.P., Ellingsen J.E., Haugen H.J. (2009). Titanium implant surface modification by cathodic reduction in hydrofluoric acid: Surface characterization and in vivo performance. J. Biomed. Mater. Res. Part A.

[34] Roodman G.D. (1996). Advances in bone biology: The osteoclast. Endocr. Rev..

[35] Boyle W.J., Simonet W.S., Lacey D.L. (2003). Osteoclast differentiation and activation. Nature.

[36] Drake F.H., Dodds R.A., James I.E., Connor J.R., Debouck C., Richardson S., Lee-Rykaczewski E., Coleman L., Rieman D., Barthlow R. (1996). But not cathepsins B, L, or S, is abundantly expressed in human osteoclasts. J. Biol. Chem..

[37] Väänänen H.K., Karhukorpi E.K., Sundquist K., Wallmark B., Roininen I., Hentunen T., Tuukkanen J., Lakkakorpi P. (1990). Evidence for the presence of a proton pump of the vacuolar H^+^-ATPase type in the ruffled borders of osteoclasts. J. Cell Biol..

[38] Engsig M.T., Chen Q.J., Vu T.H., Pedersen A.C., Therkidsen B., Lund L.R., Henriksen K., Lenhard T., Foged N.T., Werb Z. (2000). Matrix metalloproteinase 9 and vascular endothelial growth factor are essential for osteoclast recruitment into developing long bones. J. Cell Biol..

[39] Pfaffl M.W. (2001). A new mathematical model for relative quantification in real-time RT-PCR. Nucleic Acids Res..

